# Antennal Transcriptome Profiling Reveals Gustatory Receptors Associated with Pollen Foraging Preferences in *Apis mellifera*

**DOI:** 10.3390/ani16132067

**Published:** 2026-07-04

**Authors:** Qiyan Su, Yu Zhang, Chang Song, Lina Guo, Yuan Guo

**Affiliations:** 1 College of Animal Science, Shanxi Agricultural University, Jinzhong 030801, China; yanqisu@163.com (Q.S.); 15183537865@163.com (Y.Z.); sconnie30@163.com (C.S.); 2 Shanxi Key Laboratory of Animal Nutrition and Feed Development, College of Animal Science, Shanxi Agricultural University, Jinzhong 030801, China; 3 College of Horticulture, Shanxi Agricultural University, Jinzhong 030801, China

**Keywords:** *Apis mellifera*, antennal transcriptome, gustatory receptor genes, pollen foraging, differential expression analysis

## Abstract

Gustatory perception in honeybees is a key determinant of foraging decisions and pollen source selection. However, the molecular mechanisms underlying this sensory discrimination remain poorly understood. To investigate these mechanisms during the collection of pollen from different floral sources, this study utilized antennae from worker bees foraging on pear and rapeseed pollen, and non-pollen-foraging workers as controls. Illumina high-throughput transcriptome sequencing was employed to identify differentially expressed genes (DEGs), perform functional annotation, and characterize gustatory receptor (GR) genes. Compared with the control group, 583 DEGs and 516 DEGs were identified in pear-pollen and rapeseed-pollen foragers, respectively, whereas only 73 DEGs were detected between the two pollen-foraging groups. Several DEGs were associated with chemosensory perception, signal transduction, energy metabolism, and immune responses. Notably, genes involved in membrane-associated signaling and stimulus response exhibited differential expression patterns among foraging groups, suggesting adaptive molecular responses to distinct floral resources. Gene Ontology (GO) analysis indicated that DEGs were primarily associated with cellular processes, membrane components, and binding functions. Kyoto Encyclopedia of Genes and Genomes (KEGG) pathway enrichment highlighted significant involvement in phagosome, phosphatidylinositol signaling system, oxidative phosphorylation, and extracellular matrix–receptor interaction. Notably, seven GR-related genes were identified in the antennal transcriptome, including five known GR genes and two novel candidates, all with complete open reading frames. Four of these genes featured the canonical seven-transmembrane domain structure of insect GRs. Phylogenetic analysis, in addition to the known sugar receptors AmelGR43a, AmelGR64f, and AmelGR64f-X1, based on GRs from *Apis mellifera* and Drosophila melanogaster suggested that AmelGR28b, AmelGR10, AmelGR12, and AmelGR13 may belong to the bitter taste receptor family. Quantitative real-time PCR (qRT-PCR) validation demonstrated that the expression patterns of the selected seven DEGs were consistent with the RNA-seq results. This study reveals differential expression patterns and potential functional divergence of gustatory receptor genes in *Apis mellifera* during pollen collection from different floral sources. It provides important molecular evidence for understanding how honeybees accurately recognize and preferentially forage specific pollen sources via gustatory perception, and offers valuable theoretical and practical insights for honeybee behavioral ecology and crop pollination management.

## Simple Summary

Honeybees rely on their antennae to detect chemical signals from flowers, helping them choose suitable pollen and nectar sources during foraging. However, the molecular basis underlying how honeybees recognize different floral resources remains poorly understood. In this study, we compared gene expression in the antennae of western honeybees (*Apis mellifera*) collecting pear pollen, rapeseed pollen, and no pollen. Transcriptome sequencing identified hundreds of genes that were differently expressed among the groups, many of which were associated with sensory perception, signal transmission, metabolism, and environmental response. We further identified seven gustatory receptor genes related to taste perception in honeybee antennae. Three genes were associated with sugar detection, while four genes were likely involved in bitter substance perception. These genes may help honeybees distinguish floral resources and avoid harmful compounds during foraging. The expression patterns of these genes were further confirmed using quantitative real-time PCR analysis. This study improves our understanding of how honeybees recognize and select different pollen sources at the molecular level. The findings provide valuable information for honeybee behavioral research, pollination biology, and the sustainable management of agricultural pollination resources.

## 1. Introduction

Bees, as indispensable pollinators in natural ecosystems, play a crucial role in maintaining ecological balance and promoting agricultural production [1,2,3]. Among them, *Apis mellifera* dominates the global apiculture industry due to its high foraging efficiency, strong adaptability, and high honey yield [4,5]. The foraging behavior of honeybees relies heavily on a well-developed sensory system for acquiring and identifying floral resource information. As one of the most important sensory organs, the antenna plays a key role in environmental sensing, encompassing odor recognition, gustatory perception, and other forms of sensory input [6,7,8]. The surface of the antenna is densely covered with various sensilla, including olfactory and gustatory receptors, which can effectively detect complex chemical signals released by nectar and pollen, thereby guiding bees to recognize and select suitable floral resources with high precision [9].

Floral resources provide bees with essential nectar and pollen for survival. Different plant species exhibit significant variations in nectar composition, volatile odors, and nutritional content [10], all of which directly influence the foraging behavior and feeding preferences of bees [11,12]. Through long-term co-evolution, honeybees have developed a highly sensitive chemosensory system. Previous studies have demonstrated that multiple gene families closely related to chemosensory functions are present in honeybee antennae, including odorant-binding proteins (OBPs), olfactory receptors (ORs), ionotropic receptors (IRs), and gustatory receptors (GRs) [13]. Among these, GR genes play a key role in sensing sugars, amino acids, and other non-volatile chemical substances, profoundly influencing honeybee foraging behavior [14,15,16].

In recent years, the rapid development of transcriptome sequencing (RNA-seq) has substantially advanced the understanding of honeybee chemosensory systems. Antennal transcriptome analyses have revealed that gene expression profiles dynamically change with worker behavioral specialization and environmental adaptation [8]. Recent studies in *Apis cerana cerana* further demonstrated that GR genes exhibit differential expression patterns when workers forage on different pollen resources, suggesting that floral chemical composition may influence peripheral sensory regulation [9]. Moreover, functional studies have shown that insect chemoreceptors possess considerable molecular plasticity, with individual receptors capable of recognizing multiple chemical signals [17].

Despite these advances, previous studies have mainly focused on the identification of honeybee GR genes or their evolutionary characteristics, whereas their transcriptional regulation under different pollen-foraging conditions remains poorly understood. Particularly in *Apis mellifera*, comparative studies investigating GR gene expression when collecting pollen from different floral sources are scarce. However, it remains unknown whether different floral resources, through their unique chemical compositions, directly induce the differential expression of receptor-related genes in the antennae, which in turn finely tunes honeybee foraging strategies and floral preferences [1,18]. Therefore, comparing the antennal transcriptome data of A. mellifera collecting pollen from different floral sources is of great importance for elucidating the molecular mechanisms of floral perception.

Based on this, the present study investigated *Apis mellifera* collecting pollen from diverse floral sources. We systematically performed transcriptome sequencing and bioinformatics analysis on their antennal tissues to identify differentially expressed genes, with a particular focus on the expression characteristics of gustatory receptor genes. Furthermore, combined with Gene Ontology (GO) functional annotation and Kyoto Encyclopedia of Genes and Genomes (KEGG) pathway analysis, we explored the roles of these genes in relevant biological processes and signaling pathways. Ultimately, this study provides a theoretical basis for a deeper understanding of the molecular mechanisms underlying floral recognition and honeybee foraging behavior, while also offering scientific insights for the rational utilization of floral resources and the sustainable development of the apiculture industry.

## 2. Materials and Methods

### 2.1. Experimental Insects

*Apis mellifera* samples were collected from 23 March to 29 March 2022, at the ecological pear orchard base in Hongzhi Town, Yanhu District, Yuncheng City (geographical coordinates: 35.0151° N, 110.99827° E). The orchard spanned approximately 54 hm^2^, dominated by pear trees as the primary cultivated species. Rapeseed was planted about 1000 m away from the pear orchard, covering 0.2 hm^2^, with a competing plant proportion of 0.4%. Six Langstroth hives were established with bee colonies. Each colony was adjusted to contain approximately 3000–3500 worker bees, four combs, one queen and a small number of larvae, with negligible initial pollen and nectar stores. When approximately 25% of the pear blossoms were in bloom, the colonies were transferred to the orchard. The six hives were then positioned in a single row between the pear orchard and the rapeseed field. The following day, between 11:00 and 15:00, worker bees were collected at the hive entrance and categorized into three groups: bees carrying pear pollen, bees carrying rapeseed pollen, and a control group carrying no pollen. Bees carrying pear pollen and rapeseed pollen were distinguished according to the color and morphology of pollen pellets on the corbiculae. Pollen identity was further verified microscopically using representative samples. Worker bees without visible pollen loads on both hind legs were assigned to the control group. Due to the extremely small size of individual honey bee antennae and the correspondingly low RNA yield, a pooling strategy was employed. For each treatment group, a total of 600 bees were collected and divided into three independent biological replicates. Each biological replicate consisted of antennae pooled from 200 adult forager bees. This strategy was designed to minimize individual-level variation and to ensure that sufficient high-quality RNA could be obtained for transcriptome sequencing and downstream qPCR validation. After collection, all antennae were immediately dissected on ice, rapidly frozen in liquid nitrogen, and stored at −80 °C for subsequent use. To minimize environmental variation, all samples were collected under sunny weather conditions with temperatures ranging from 18–24 °C and wind speeds below level 3. Sampling was conducted within the same daily time window (11:00–15:00). Colonies were located at equal distances from major floral resources to reduce spatial bias in foraging opportunities.

### 2.2. RNA Extraction, Library Construction, and Transcriptome Sequencing

Total RNA was extracted from the antennae of worker bees using TRIzol reagent (Thermo Fisher Scientific, San Diego, CA, USA, Cat. No. 15596018). Based on the pollen carried, samples were divided into three groups: pear pollen collection (LC), rapeseed pollen collection (YC), and non-pollen-foraging group (CC). Each group included three biological replicates (LC1–LC3, YC1–YC3, CC1–CC3), and each replicate consisted of antennae from 200 worker bees. RNA purity and concentration were measured with a NanoDrop 2000 spectrophotometer (Thermo Fisher Scientific, Wilmington, DE, USA), and RNA integrity was assessed with an Agilent 2100 Bioanalyzer (Agilent Technologies, Santa Clara, CA, USA; Cat. No. 5067-1511). Qualified RNA samples were subjected to transcriptome sequencing by Biomarker Technologies Co., Ltd. (Beijing, China) The RNA samples remaining after sequencing were also used for subsequent qRT-PCR experiments.

mRNA was enriched using oligo(dT) magnetic beads (Thermo Fisher Scientific, San Diego, CA, USA), followed by fragmentation. Using the mRNA as a template, first- and second-strand cDNA were synthesized and purified. The resulting double-stranded cDNA was end-repaired, A-tailed, and ligated with an adaptor. Fragment size selection was performed using AMPure XP beads (Beckman Coulter, Beverly, MA, USA), and PCR amplification was used to construct the cDNA library.

After library construction, initial quantification was performed with a Qubit 3.0 fluorometer (MAN0016388 Rev. A.0) (concentration ≥ 1 ng/μL). Insert size distribution was analyzed using the Qsep400 high-throughput system. Libraries with expected insert sizes were further quantified by qRT-PCR (effective concentration > 2 nmol/L). Qualified libraries were sequenced on the Illumina NovaSeq 6000 platform using the paired-end 150 bp (PE150) strategy.

### 2.3. Transcriptome Data Analysis

Raw reads were initially filtered to remove adapters, poly-N sequences, and low-quality bases, yielding clean reads. For downstream analyses, only clean reads exhibiting a Q30 values exceeding 95% were retained. Differentially expressed genes (DEGs) were identified using DESeq2 v1.30.1, with significance defined by a |log2FoldChange| ≥ 1.5 and a false discovery rate (FDR) < 0.01. Clean reads, obtained after filtering raw sequencing data, were aligned to the reference genome of *Apis mellifera* using HISAT2 v2.2.1 (hisat2-2.2.1) with default parameters. These reads were mapped to the honeybee genome to quantify gene expression levels. For functional annotation of differentially expressed genes (DEGs), Gene Ontology (GO) analysis was performed, categorizing annotated genes into three categories: biological process, molecular function, and cellular component. Kyoto Encyclopedia of Genes and Genomes (KEGG) pathway analysis was also conducted to map genes to metabolic pathways, thereby elucidating differences in molecular functions and metabolic pathways under different foraging conditions (pear pollen, rapeseed pollen, and no pollen).

### 2.4. Screening and Analysis of Gustatory Receptor Genes

The antennal transcriptome data of *Apis mellifera* were analyzed, and genes annotated as GR (gustatory receptor) were screened using keyword searches. These GR gene sequences were subjected to BLAST (https://blast.ncbi.nlm.nih.gov/Blast.cgi, accessed on 20 February 2026) analysis in the NCBI database to identify the most homologous species, gene names, and accession numbers.

Open reading frames (ORFs) of the GR genes were predicted using the online ORF Finder tool (https://www.ncbi.nlm.nih.gov/orffinder/, accessed on 14 March 2026). Subsequently, amino acid sequences of candidate GRs were analyzed using TMHMM 2.0 (https://services.healthtech.dtu.dk/services/TMHMM-2.0/, accessed on 14 March 2026) to predict transmembrane domains.

Candidate GR genes were identified based on BLAST annotation, ORF prediction, and transmembrane-domain analysis. Predicted transmembrane domains and conserved C-terminal motifs were used as supporting evidence for GR annotation.

### 2.5. Phylogenetic Analysis of Gustatory Receptor Genes

To investigate the evolutionary relationships between the seven *Apis mellifera* GRs (Amel GRs) and GRs from other Hymenoptera insects, amino acid sequences of GRs from four species (*Apis cerana*, *Apis dorsata*, *Apis laboriosa*, and *Apis florea*) were retrieved from the NCBI database.

Multiple sequence alignment was performed using MEGA version 12.0., and a phylogenetic tree was constructed using the neighbor-joining (NJ) method in MEGA 7.0. The Amel GRs were subsequently named accordingly.

Furthermore, to analyze the functional characteristics of these seven Amel GRs, their amino acid sequences were compared with 53 GRs from Drosophila melanogaster, and a phylogenetic tree was constructed to infer their functional classification.

### 2.6. Quantitative Real-Time PCR (qRT-PCR) Analysis

To validate the reliability of the RNA-seq data, quantitative real-time PCR (qRT-PCR) was performed to analyze the relative expression levels of seven GR-related genes identified in the antennal transcriptome of worker bees foraging on pear and rapeseed flowers.

Total RNA was reverse-transcribed into cDNA, which was used as the template for qRT-PCR. Amplification was conducted using the TB Green^®^ Premix Ex Taq™ (Tli RNaseH Plus) (Takara Bio Inc., Kusatsu, Japan) kit according to the manufacturer’s instructions.

The qRT-PCR reaction mixture (10 μL total volume) contained 5 μL TB Green^®^ Premix Ex Taq™, 0.5 μL of each forward and reverse primer (10 μM), 500 ng cDNA template, and nuclease-free water.

The amplification conditions were as follows: initial denaturation at 95 °C for 3 min, followed by 40 cycles of 95 °C for 5 s, 57 °C for 30 s, and 72 °C for 30 s. Melting curve analysis was performed at 95 °C for 10 s and 52 °C for 5 s.

The *Apis mellifera Arp1* gene (GenBank accession number: NM_001185145.1) was used as the internal reference gene. Each experimental group included three biological replicates and three technical replicates. Relative gene expression levels were calculated using the 2^−ΔΔCt^ method. Primer sequences are listed in Table 1.

## 3. Results

### 3.1. Antennal Transcriptome of Worker Apis mellifera

To characterize the gene expression profiles of antennae from worker *Apis mellifera* collecting pollen from different floral sources, Illumina sequencing technology was employed to sequence antennal samples. In this study, a total of nine cDNA libraries were constructed, including three antennal libraries from workers collecting pear pollen (LC1, LC2, and LC3), three from workers collecting rapeseed pollen (YC1, YC2, and YC3), and three from workers not collecting pollen (CC1, CC2, and CC3).

Through eukaryotic reference-based transcriptome (RNA-seq) analysis of the nine samples, a total of 61.59 Gb of clean data was obtained. The clean data for each sample exceeded 6.04 Gb, and the GC content ranged from 37.00% to 38.88%. The percentage of Q30 bases for all samples was above 95.61%, indicating that the filtered data were of high quality and suitable for subsequent bioinformatics analyses (Table 2).

### 3.2. Alignment of Antennal Transcriptome Sequences to the Reference Genome of Apis mellifera

As shown in Table 3, the mapping efficiency of reads from each sample to the reference genome ranged from 87.63% to 93.94%, while the proportion of reads uniquely mapped to the reference genome ranged from 86.21% to 92.08%. Under normal conditions, if samples are free from contamination, the mapping rate is typically above 70%.

In each sample, more than 78% of the reads were mapped to exonic regions. The proportion of reads mapped to intronic regions was consistently higher than that mapped to intergenic regions. The percentage of reads mapped to intergenic regions ranged from a maximum of 10.6% (CC1) to a minimum of 9.84% (CC3), with only minor differences between samples.

### 3.3. Identification of Differentially Expressed Genes (DEGs)

**Figure 1:**
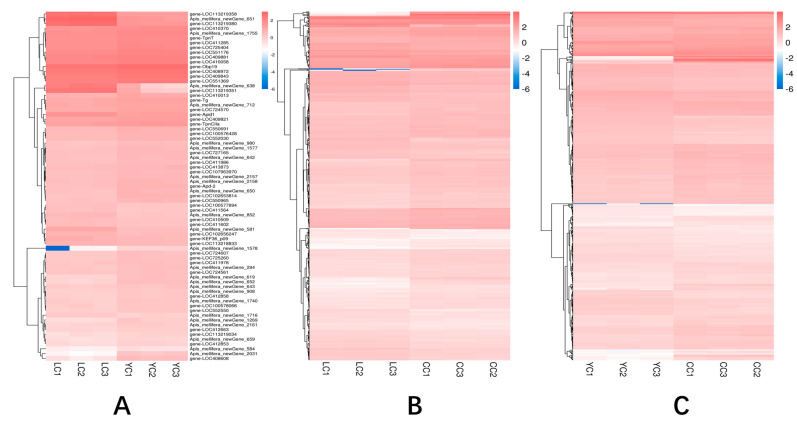
Clustering heatmap of differentially expressed genes in the antennae of *Apis mellifera* collected with different pollens. Note: (**A**), LC vs. YC; (**B**), LC vs. CC; (**C**), YC vs. CC. The color scale represents normalized log2(FPKM + 1) expression values.

Cluster heatmap analysis of differentially expressed genes (Figure 1) revealed significant differences in gene expression patterns among different treatment groups, while biological replicates within the same group clustered closely together, indicating good reproducibility of the experimental data. Red indicates relatively higher expression and blue indicates relatively lower expression based on normalized log2(FPKM + 1) values.

**Figure 2:**
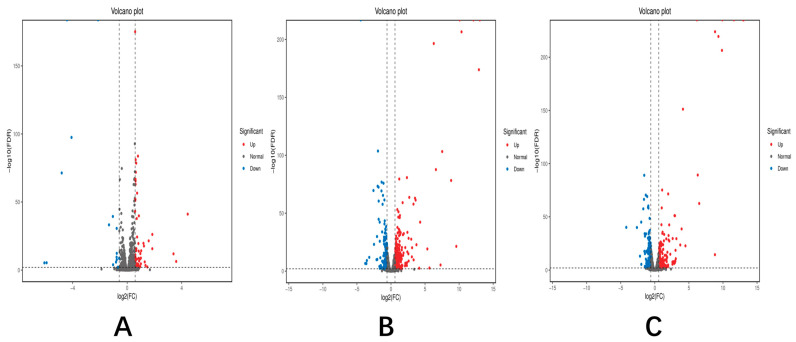
Volcano plot of differentially expressed genes in the antennae of *Apis mellifera* collected with different pollens. Note: (**A**), LC vs. YC; (**B**), LC vs. CC; (**C**), YC vs. CC.

Subsequently, differential expression analysis was performed using DESeq2 v1.30.1. The results showed that a total of 73 DEGs were identified in the LC vs. YC comparison, including 57 upregulated and 16 downregulated genes. In the LC vs. CC comparison, 583 DEGs were identified, of which 357 were upregulated and 226 were downregulated. In the YC vs. CC comparison, 516 DEGs were identified, including 282 upregulated and 234 downregulated genes (Figure 2).

### 3.4. Functional Classification of Differentially Expressed Genes Between Worker Honeybees Collecting Pear and Rapeseed Pollen

**Figure 3:**
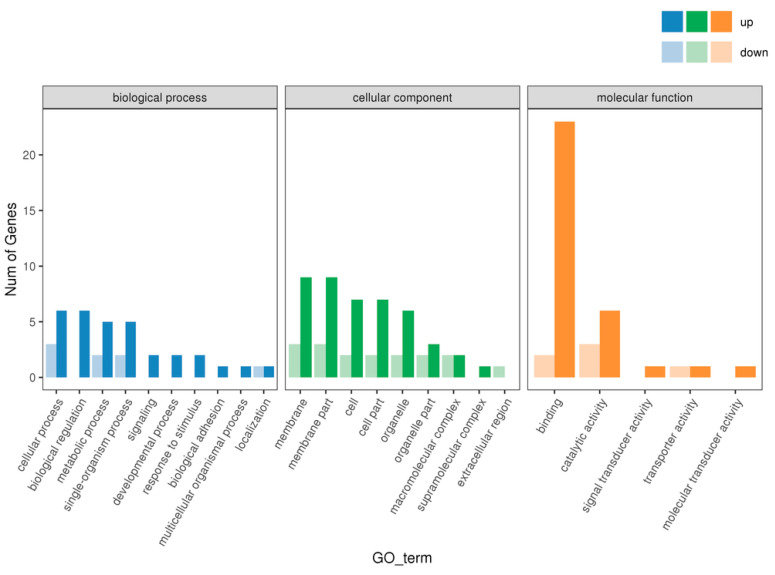
GO functional annotation of differentially expressed genes between the antennae transcriptomes of *Apis mellifera* collecting pear pollen and rapeseed pollen.

Gene Ontology (GO) analysis classified the differentially expressed genes (DEGs) into 30 functional groups, which were further grouped into three major categories: biological process, molecular function, and cellular component (Figure 3). These categories describe the biological processes in which gene products participate, their molecular functions, and their cellular localization, respectively.

Among the three GO categories, the most enriched terms were cellular process, membrane, and binding. The enrichment of membrane and binding categories strongly suggests active chemosensory signal perception and transduction within antennal tissues. Concurrently, genes related to broader cellular processes may underpin the neuronal activity required for accurate pollen discrimination.

**Figure 4:**
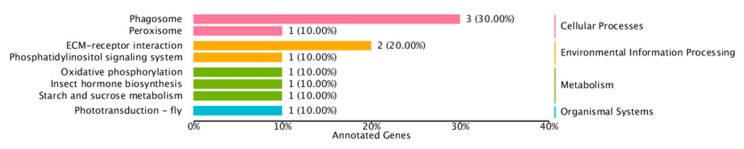
Functional annotation of KEGG for differentially expressed genes between the antennae transcriptomes of *Apis mellifera* collecting pear pollen and rapeseed pollen.

KEGG pathway analysis indicated that DEGs were significantly enriched in pathways related to phagosome (three genes), extracellular matrix–receptor interaction (two genes), oxidative phosphorylation (one gene), and phosphatidylinositol signaling (one gene) (Figure 4). The enrichment of the phosphatidylinositol signaling pathway and ECM–receptor interactions indicates that intracellular signal transduction and receptor-mediated communication are likely involved in processing chemical cues derived from different pollen sources.

### 3.5. Sequence Analysis of Candidate Gustatory Receptor Genes in Worker Apis mellifera

Analysis of the antennal transcriptome of *Apis mellifera* identified a total of seven GR (gustatory receptor) genes. Based on BLAST alignment, open reading frame (ORF) prediction, and transmembrane domain analysis, all seven genes were confirmed to possess complete ORFs.

The longest ORF of GR10 was 2514 bp, showing 100% similarity to the gustatory receptor protein 10 transcript variant X2 of *Apis mellifera* (accession number: XM_006567110.3). The longest ORF of LOC725483 was 1425 bp, showing 100% similarity to gustatory receptor 43a of *Apis mellifera* (XM_016913387.2). The longest ORF of LOC413684 was 1413 bp, showing 100% similarity to gustatory receptor protein 64f of *Apis mellifera* (XM_397125.7), and it contained seven transmembrane domains. The longest ORF of LOC727431 was 1458 bp, showing 100% similarity to gustatory receptor protein 64f transcript variant X1 (XM_016912472.2). The longest ORF of LOC102655663 was 1299 bp, showing 100% similarity to gustatory receptor protein 28b (XM_016918080.2), and it contained seven transmembrane domains. The longest ORF of LOC100577743 was 1254 bp, showing 91.26% similarity to gustatory receptor protein 28b of *Apis dorsata* (XM_397125.7), and it also contained seven transmembrane domains. The longest ORF of LOC107964982 was 1362 bp, showing 96.30% similarity to a gustatory receptor protein 32a-like of *Apis dorsata* (XM_043933721.1), and it contained seven transmembrane domains.

### 3.6. Phylogenetic Analysis of Gustatory Receptors (Amel GRs)

**Figure 5:**
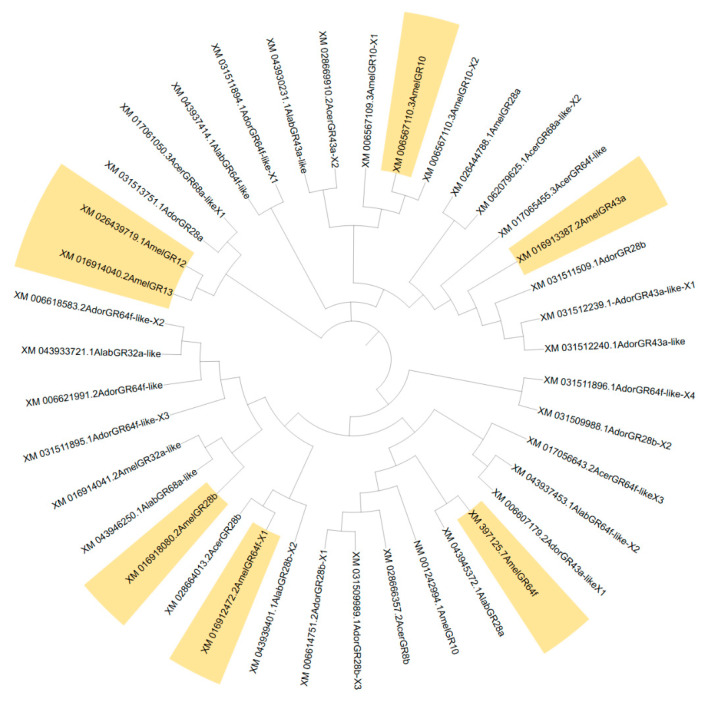
Phylogenetic trees were constructed for the candidate gustatory receptor of *Apis mellifera* (Amel GRs) and the gustatory receptor (GRs) of other hymenopteran insects.

A phylogenetic tree based on amino acid sequences was constructed using the neighbor-joining method (Figure 5). The results showed that several *Apis mellifera* GRs exhibited clear homologous relationships with GRs from other *Apis species.* Specifically, AmelGR64f-X1 was homologous to AcerGR28b (*Apis cerana cerana*) and clustered with AlabGR28a-X2 (*Apis laboriosa*), while AmelGR64f showed homology with AlabGR28a. AmelGR28b grouped with AlabGR68a-like and AmelGR32a-like within the same clade. In addition, AmelGR12 and AmelGR13 clustered with AdorGR28a (*Apis dorsata*) and AcerGR68a-like-X1, whereas AmelGR43a grouped with AdorGR28b and related homologs.

BLAST analysis of nucleotide sequences further confirmed that AmelGR43a, AmelGR64f, and AmelGR64f-X1 belong to the sugar receptor subfamily.

**Figure 6:**
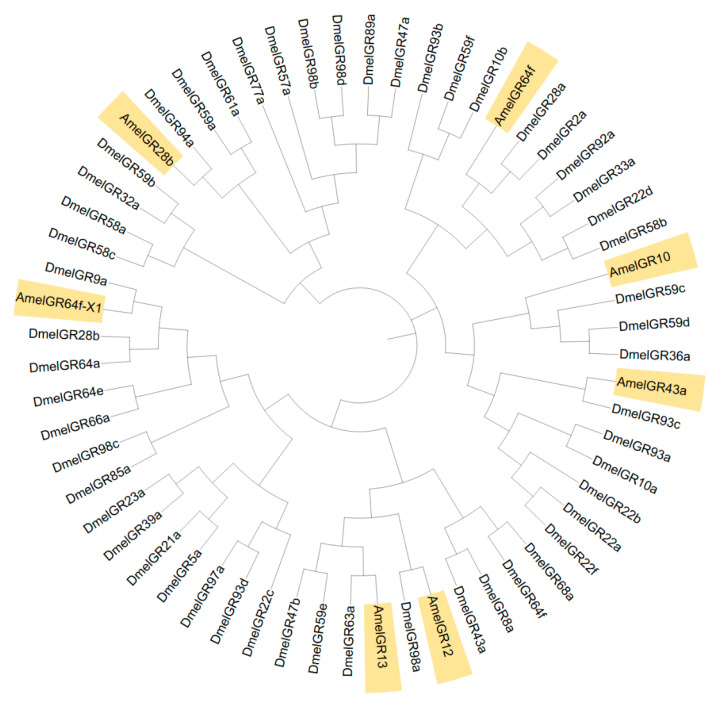
Phylogenetic tree of Amel GRs from *Apis mellifera* and GRs from *Drosophila melanogaster* based on amino acid sequence constructed using neighbor-joining method.

To further investigate functional classification, an additional phylogenetic tree was constructed using amino acid sequences of 53 gustatory receptors from Drosophila melanogaster together with the seven GRs identified in this study (Figure 6). The results showed that AmelGR10 clustered with DmelGR59c, DmelGR59d, and DmelGR36a, while AmelGR28b grouped with DmelGR94a, DmelGR59a, and DmelGR61a. AmelGR64f clustered with DmelGR2a and DmelGR28a. AmelGR12 and AmelGR13 were closely related to DmelGR98a and DmelGR63a, respectively, and clustered within the same clade as DmelGR59e and DmelGR47b. AmelGR43a showed homology to DmelGR93c, whereas AmelGR64f-X1 clustered with AmelGR9a and grouped with DmelGR64a and DmelGR28b.

Based on these phylogenetic relationships, AmelGR10, AmelGR28b, AmelGR12, and AmelGR13 are likely members of the bitter taste receptor family.

### 3.7. qRT-PCR Validation of Candidate Gustatory Receptor Genes

**Figure 7:**
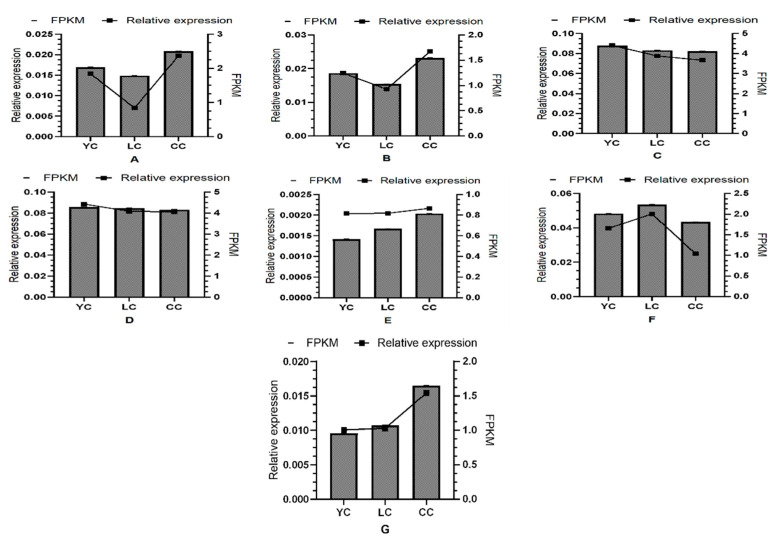
Relative expression profiles of seven *Apis mellifera* gustatory receptor (GR) genes determined by qRT-PCR. Note: (**A**): AmelGR64f-X1. (**B**): AmelGR28b. (**C**): AmelGR43a. (**D**): AmelGR64f. (**E**): AmelGR12. (**F**): AmelGR13. (**G**): AmelGR10. qRT-PCR results are shown for trend validation only; no statistical comparisons among groups are indicated.

To further validate the reliability of the transcriptome data, selected gustatory receptor (GR) genes were analyzed by qRT-PCR. The results demonstrated that the expression patterns were consistent with RNA-seq data (FPKM values), supporting the reliability of the transcriptome analysis (Figure 7). Expression trends were generally consistent with RNA-seq results, supporting the reliability of transcriptome sequencing. The qRT-PCR analysis was used to qualitatively validate the consistency of expression trends with the RNA-seq data, and no statistical significance among treatment groups is claimed based on these results.

## 4. Discussion

Based on the antennal transcriptome data of *Apis mellifera*, this study systematically analyzed genes annotated as gustatory receptors (GRs) in the pear pollen collection group, the rapeseed pollen collection group, and the control group, followed by comprehensive bioinformatics analysis and functional annotation. Our results identified seven GR genes in the antennae of *Apis mellifera.* Additionally, some GR genes may remain unidentified, possibly because gustatory receptor genes are typically expressed in specific sensory cells and at relatively low levels, making them prone to underestimation in transcriptome sequencing [19,20]. GO enrichment analysis revealed that differentially expressed genes were significantly enriched in biological processes, including cellular processes, metabolic processes, and response to stimulus. The strong enrichment in “response to stimulus” indicates that antennal tissues play a crucial role in sensing and recognizing diverse environmental cues. The antenna’s surface is densely covered with various types of sensilla, which house olfactory receptor neurons. These neurons are adept at detecting environmental chemical signals and transmitting them to the insect’s central nervous system via neural pathways, thereby fundamentally regulating insect behavior [21]. Zhao et al. [22] reported tissue-specific expression of chemosensory genes in *Apis cerana* drones, indicating that antennae possess highly specialized molecular machinery for chemical perception, which is consistent with our transcriptomic observations.

Cellular component analysis indicated that the DEGs were significantly enriched in membrane-related categories, including membrane and membrane part. Given that most insect chemosensory receptors are transmembrane proteins [23], this enrichment strongly correlates with the chemosensory function of antennae. Furthermore, the enrichment of organelle-related genes suggests robust metabolic activity in antennal cells, which likely supports the energy and material requirements for receptor protein biosynthesis and signal transduction. Additionally, a substantially larger number of DEGs were identified between the pear pollen group and the control group than between the rapeseed pollen group and the control group. This difference likely reflects the more complex nutritional composition and secondary metabolite profile of pear pollen [10], leading to broader transcriptional regulation of antennal sensory and metabolic pathways. In contrast, the relatively small number of DEGs between the pear and rapeseed pollen groups suggests that both groups shared similar physiological states associated with active pollen foraging [12], while the major transcriptional differences occurred primarily between pollen-foraging and non-pollen-foraging workers. In terms of molecular function, the significant enrichment of genes associated with binding and catalytic activity indicates a high abundance of ligand-binding proteins in the antennae. These proteins likely encompass gustatory receptors, olfactory receptors, and odorant-binding proteins, which collectively facilitate chemical signal recognition. Concurrently, genes linked to catalytic activity-related genes may play roles in the metabolism or degradation of chemical signal molecules, thereby maintaining the antennal chemosensory system’s normal operation.

KEGG pathway analysis indicated that DEGs were mainly enriched in pathways including phagosome, ECM–receptor interaction, phosphatidylinositol signaling system, and oxidative phosphorylation. The significant enrichment of the phagosome pathway suggests that antennal tissues may possess certain immune defense mechanisms. Given that insect antennae are perpetually exposed to the external environment and susceptible to microbial or pathogenic encounters, phagosome-related pathways likely support physiological homeostasis by facilitating the clearance of foreign substances [24].

ECM–receptor interaction and phosphatidylinositol signaling system are typical signal transduction pathways that play important roles in cellular responses to external stimuli [25,26]. In insect sensory systems, these pathways are essential for chemical signal recognition and for the efficient transmission of neural signals. Therefore, the enrichment of these pathways may reflect complex regulatory mechanisms underlying chemical perception in the antennae. Additionally, the enrichment of the oxidative phosphorylation pathway indicates a high level of energy metabolism in antennal tissues. Considering that chemosensory processes—including neural signal transmission and receptor protein synthesis—require substantial energy consumption, efficient energy metabolism is critical for maintaining normal antennal function. The enrichment of starch and sucrose metabolism pathways may also be related to the efficient utilization of sugar substances in nectar by honeybees. Although oxidative phosphorylation and phosphatidylinositol signaling pathways emerged from the enrichment analysis, the sparse representation of DEGs within each suggests a cautious interpretation. Therefore, while these pathways might indicate nascent physiological responses to pollen foraging, their true functional importance cannot be conclusively determined without additional experimental validation.

Phylogenetic analysis revealed that AmelGR10 clustered closely with DmelGR59c, DmelGR59d, and DmelGR36a. Given that the GR59 family comprises established bitter receptors [27], based on phylogenetic clustering with experimentally characterized *Drosophila* GRs, AmelGR10 is tentatively assigned as a bitter receptor. Nevertheless, this functional prediction necessitates further experimental validation, ideally employing heterologous expression systems or electrophysiological assays.

Similarly, AmelGR28b exhibits homology to DmelGR94a and clusters with DmelGR59a and DmelGR61a, which mainly consist of bitter receptor members, implying its involvement in detecting bitter compounds. AmelGR12 and AmelGR13 also form homologous branches with Drosophila bitter receptors, reinforcing their prospective roles in bitter substance perception. But they should be regarded as putative bitter receptors rather than functionally confirmed receptors. In insects, bitter receptors generally mediate the detection of plant secondary metabolites or toxic compounds, enabling avoidance of harmful ingestants [28,29].

During long-term evolution, plants have developed numerous defensive chemical compounds [30], such as alkaloids, phenolics, and terpenoids, many of which exhibit strong bitter characteristics [31]. Therefore, bitter receptors in honeybees may help them identify and avoid potentially toxic floral sources in complex foraging environments, thereby improving foraging safety. In addition to bitter perception, CO_2_ detection also plays an important role in regulating honeybee social behavior, including nest ventilation and colony communication [32]. In Drosophila, GR21a and GR63a jointly form a CO_2_ detection system that senses changes in environmental CO_2_ concentrations [33]. In this study, although AmelGR13 clustered with DmelGR63a in the phylogenetic analysis, no functional evidence is available in the present study to support its involvement in CO_2_ perception. Therefore, its biological function requires further experimental validation.

Although substantial progress has been made in understanding insect gustatory receptor families in recent years, research on GRs in non-model insects remains relatively limited compared with Drosophila [34,35]. Through long-term evolution, *Apis mellifera* has developed a highly sophisticated chemosensory system, enabling it to accurately recognize chemical signals released by different floral sources and selectively forage specific plants [36].

Compared with the recent antennal transcriptome study of *Apis cerana*, which also identified sugar and bitter receptor subfamilies associated with pollen foraging, the present study revealed a highly conserved GR repertoire in *Apis mellifera* while demonstrating species-specific expression patterns under different pollen sources. In addition, our results indicate that antennal transcriptomic responses are dominated by the transition between pollen-foraging and non-pollen-foraging states rather than differences between floral species. These findings extend previous observations and provide new evidence for the molecular basis of floral resource recognition in western honeybees. These findings demonstrate an association between GR gene expression and pollen source selection in honeybees rather than a direct causal relationship. Functional studies, including gene knockdown, gene editing, electrophysiological analyses, and behavioral assays, will be required to verify the biological roles of these receptors in pollen preference.

While this study provides valuable insights into honeybee foraging behavior, we acknowledge certain limitations. First, regarding the experimental design, only two pollen sources (pear and rapeseed) were examined, which limits the generalizability of our conclusions. Nevertheless, because both species are dominant early-spring flowering crops in the local agroecosystem—and honeybees naturally exhibit a clear foraging preference for rapeseed while largely avoiding pear—this strong behavioral contrast provided an ideal ecological context for investigating the molecular basis of pollen selection. Second, although our transcriptomic profiling reveals a strong association between GR gene expression and pollen selection, it does not yet establish a direct causal relationship. The present study was primarily designed to screen candidate GRs rather than to functionally verify their roles. Third, our analysis focused exclusively on antennal tissues. While antennae are the primary chemosensory organs that initially encounter and evaluate floral cues, gustatory receptors are also abundantly expressed in other contact-chemosensory appendages, such as the proboscis and tarsi. Thus, focusing solely on antennae provides only a partial view of the gustatory landscape. Despite these limitations, the candidate GRs identified here provide a crucial foundation and clear targets for future research. Moving forward, a comprehensive approach will be required to build upon these findings: ecologically, incorporating a broader range of floral resources will determine whether these expression patterns reflect pollen-specific responses or general mechanisms underlying floral discrimination; anatomically, integrating transcriptomic analyses across multiple sensory organs (antennae, proboscis, and tarsi) will help comprehensively map the GR-mediated networks; and mechanistically, functional experiments—including RNAi-mediated knockdown, CRISPR/Cas9 editing, heterologous expression, electrophysiology, and behavioral assays—will be essential to definitively establish causality and confirm the biological roles of these receptors driving honeybee foraging preferences.

## 5. Conclusions

Antennal transcriptome analysis of *Apis mellifera* foragers collecting pear and rapeseed pollen identified seven gustatory receptor (GR) genes. Among these, AmelGR43a, AmelGR64f, and AmelGR64f-X1 are known sugar receptors, while AmelGR28b, AmelGR10, AmelGR12, and AmelGR13 are likely bitter receptors, suggesting foraging preference may be linked to GR function. Their precise biological functions require further experimental validation. Differentially expressed genes in the antennae participate in metabolism, immune defense, chemosensory perception, and environmental adaptation, providing a molecular basis for floral resource selection and its regulation in honeybees.

## Figures and Tables

**Table 1:** Primer Sequences.

Gene	Forward Primer Sequence	Reverse Primer Sequence
Arp1	TGCTGCACTCGTAGTTGACAATGG	ACCCTGGTGGCGTGGTCTTC
Gr10	GCCAACATTCTCGTCATCGC	TCCGCCACGGACAGATTAAC
AmelGR43a	CGCACGCGCGTATTATGTTT	TCCGCGAACGCTTAATGGTA
AmelGR64f-X1	AGCATCGCTCTAACGGATCA	CCGGCATTGCCTTAACAATGA
AmelGR64f	TGTCAACTTGCGACAGACGA	TATGATTGCTCCAGCCACCG
AmelGR28b	GCCGGTCTTGCTACGTTTTC	TAGACAGCTGCCAAACAAGT
AmelGR12	CCGTTCGACGTTGAAACCAC	TCATTACGCACACAACGGGA
AmelGR13	TGCAACCGCATTTCCATTCG	CAATTGCTCGTTCGTGTCCG

**Table 2:** Transcriptome data evaluation and statistics of the antennae of *Apis mellifera*.

Samples	Number of Clean Reads	Number of Clean Bases	G + C (%)	Q30 (%)	Samples	Number of Clean Reads
CC1	28,608,829	8,542,481,096	37.00	96.49	CC1	28,608,829
CC2	29,332,330	8,761,703,572	37.59	96.12	CC2	29,332,330
CC3	21,569,154	6,440,893,224	37.90	96.65	CC3	21,569,154
LC1	21,864,169	6,526,389,382	38.43	95.68	LC1	21,864,169
LC2	20,536,539	6,131,171,066	38.64	95.61	LC2	20,536,539
LC3	22,062,691	6,582,042,684	38.54	96.12	LC3	22,062,691
YC1	20,245,058	6,040,177,294	38.66	96.45	YC1	20,245,058
YC2	21,613,008	6,451,038,544	38.33	95.89	YC2	21,613,008
YC3	20,492,115	6,114,308,206	38.28	96.05	YC3	20,492,115

LC1–3: Three groups of worker antennal samples were collected from pear flowers, and the three groups were three replicates; YC1–3: Three groups of worker antennal samples were collected from rapeseed flowers, and the three groups were three replicates; CC1–3: Three groups of worker antennal samples without pollen collection, three groups in three replications. The same below.

**Table 3:** Alignment statistical results of antennal transcriptome sequences of *Apis mellifera* with the reference genome.

Samples	Number of Total Reads	Mapped Reads (%)	Unique Mapped Reads (%)	Exon	Intron	Intergenic
CC1	57,217,658	53,580,237 (93.64%)	52,730,340 (92.16%)	78.2	11.2	10.6
CC2	58,664,660	54,892,862 (93.57%)	54,017,588 (92.08%)	78.5	11.2	10.3
CC3	43,138,308	40,383,105 (93.61%)	39,686,682 (92.00%)	78.97	11.19	9.84
LC1	43,728,338	41,077,642 (93.94%)	39,879,720 (91.20%)	78.6	11.1	10.3
LC2	41,073,078	38,338,929 (93.34%)	37,088,235 (90.30%)	78.7	11.1	10.2
LC3	44,125,382	41,178,321 (93.32%)	39,950,910 (90.54%)	78.3	11.2	10.5
YC1	40,490,116	35,481,466 (87.63%)	34,905,499 (86.21%)	78.5	11.5	10.0
YC2	43,226,016	39,646,499 (91.72%)	39,014,604 (90.26%)	78.7	11.4	9.9
YC3	40,984,230	37,673,704 (91.92%)	37,089,031 (90.50%)	78.5	11.5	10

## Data Availability

The raw sequencing data generated in this study have been deposited in the NCBI Gene Expression Omnibus (GEO) database under accession number GSE230384. All other data supporting the findings of this study are available from the corresponding author upon reasonable request.

## References

[1] Klein A.-M., Vaissière B.E., Cane J.H., Steffan-Dewenter I., Cunningham S.A., Kremen C., Tscharntke T. (2007). Importance of pollinators in changing landscapes for world crops. Proc. R. Soc. B Biol. Sci..

[2] Aslan C.E., Liang C.T., Galindo B., Kimberly H., Topete W. (2016). The Role of Honey Bees as Pollinators in Natural Areas. Nat. Areas J..

[3] Ferenczi A.F., Szűcs I., Gáthy A.B. (2023). Evaluation of the Pollination Ecosystem Service of the Honey Bee (*Apis mellifera*) Based on a Beekeeping Model in Hungary. Sustainability.

[4] Brodschneider R. (2025). Honey, Knowledge and Development. Bee World.

[5] Olate-Olave V.R., Verde M., Vallejos L., Perez Raymonda L., Cortese M.C., Doorn M. (2021). Bee Health and Productivity in *Apis mellifera*, a Consequence of Multiple Factors. Vet. Sci..

[6] Cholé H., Merlin A., Henderson N., Paupy E., Mahé P., Arnold G., Sandoz J.C. (2022). Antenna movements as a function of odorants’ biological value in honeybees (*Apis mellifera* L.). Sci. Rep..

[7] Goulet D., Smith B., True A., Crimaldi J. (2025). Pore plate sensilla scale and distribution modulate odor capture around honey bee antennae. Sci. Rep..

[8] Ke H., Chen Y., Zhang B., Duan S., Ma X., Ren B., Wang Y. (2024). Odorant Receptors Expressing and Antennal Lobes Architecture Are Linked to Caste Dimorphism in Asian Honeybee, *Apis cerana* (Hymenoptera: Apidae). Int. J. Mol. Sci..

[9] Guo L., Song C., Zhang Y., Guo Y. (2025). Analysis of antennal transcriptome and gustatory receptor genes in *Apis cerana cerana* collecting pollens from different nectar source plants. BMC Genom..

[10] Al-Kahtani S., Taha E.K.A. (2020). Seasonal Variations in Nutritional Composition of Honeybee Pollen Loads. J. Kans. Entomol. Soc..

[11] Wright G.A., Baker D.D., Palmer M.J., Stabler D., Mustard J.A., Power E.F., Borland A.M., Stevenson P.C. (2013). Caffeine in Floral Nectar Enhances a Pollinator’s Memory of Reward. Science.

[12] Moreno E., Arenas A. (2024). Foraging task specialization in honey bees (*Apis mellifera*): The contribution of floral rewards on the learning performance of pollen and nectar foragers. J. Exp. Biol..

[13] Hansson B.S., Stensmyr M.C. (2011). Evolution of Insect Olfaction. Neuron.

[14] de Brito Sanchez M.G. (2011). Taste perception in honey bees. Chem. Senses.

[15] Robertson H.M., Wanner K.W. (2006). The chemoreceptor superfamily in the honey bee, *Apis mellifera*: Expansion of the odorant, but not gustatory, receptor family. Genome Res..

[16] Simcock N.K., Gray H., Bouchebti S., Wright G.A. (2018). Appetitive olfactory learning and memory in the honeybee depend on sugar reward identity. J. Insect Physiol..

[17] Abhishek O., Wenqing Z. (2021). Characterization of gustatory receptor 7 in the brown planthopper reveals functional versatility. Insect Biochem. Mol. Biol..

[18] Raguso R.A. (2008). Start making scents: The challenge of integrating chemistry into pollination ecology. Entomol. Exp. Appl..

[19] Joseph R.M., Carlson J.R. (2015). Drosophila Chemoreceptors: A Molecular Interface Between the Chemical World and the Brain. Trends Genet..

[20] Louise B., Réjaud A., Sandoz J.C., Carcaud J., Giurfa M., de Brito Sanchez M.G. (2021). Peripheral Taste Detection in Honey Bees: What do Taste Receptors Respond to?. Eur. J. Neurosci..

[21] Chen Z., Liu F., Liu N. (2018). Neuronal Responses of Antennal Olfactory Sensilla to Insect Chemical Repellents in the Yellow Fever Mosquito, *Aedes aegypti*. J. Chem. Ecol..

[22] Zhao S., Wu J., Geng T., Gao J., Wubie A.J., Liu J., Wang S. (2025). Transcriptome and differential expression analysis of chemosensory genes in the antennae and heads of *Apis cerana cerana* drones. J. Apic. Res..

[23] Leal W.S. (2013). Odorant Reception in Insects: Roles of Receptors, Binding Proteins, and Degrading Enzymes. Annu. Rev. Entomol..

[24] Hillyer J.F. (2016). Insect immunology and hematopoiesis. Dev. Comp. Immunol..

[25] Daley W.P., Peters S.B., Larsen M. (2008). Extracellular matrix dynamics in development and regenerative medicine. J. Cell Sci..

[26] Gilbert D.P., Pietro D.C. (2006). Phosphoinositides in cell regulation and membrane dynamics. Nature.

[27] Scott K. (2018). Gustatory Processing in *Drosophila melanogaster*. Annu. Rev. Entomol..

[28] Weiss L.A., Dahanukar A., Kwon J.Y., Banerjee D., Carlson J.R. (2011). The Molecular and Cellular Basis of Bitter Taste in Drosophila. Neuron.

[29] Wang Z., Liu D., Ma L., Cheng H., Lin C., Fu L., Chen Y., Dong X., Liu C. (2024). Genome-wide analysis of gustatory receptor genes and identification of the fructose gustatory receptor in *Arma chinensis*. Heliyon.

[30] Rering C.C., Rudolph A.B., Beck J.J. (2021). Pollen and yeast change nectar aroma and nutritional content alone and together, but honey bee foraging reflects only the avoidance of yeast. Environ. Microbiol..

[31] Mithöfer A., Boland W. (2012). Plant Defense Against Herbivores: Chemical Aspects. Annu. Rev. Plant Biol..

[32] Martin B., McVeigh A., Tsakonas C., Kumar T., Chamberlain L., Newton M.I. (2023). A Monitoring System for Carbon Dioxide in Honeybee Hives: An Indicator of Colony Health. Sensors.

[33] Jones W.D., Cayirlioglu P., Grunwald Kadow I., Vosshall L.B. (2007). Two chemosensory receptors together mediate carbon dioxide detection in *Drosophila*. Nature.

[34] Paula L., Mutis A., Palma-Millanao R., González-González A., Ceballos R., Quiroz A., Bardehle L., Hidalgo A., Torres F., Romero-López A. (2024). Comparative transcriptomic analysis of chemoreceptors in two sympatric scarab beetles, *Hylamorpha elegans* and *Brachysternus prasinus*. Comp. Biochem. Physiol. Part D. Genom. Proteom..

[35] Paula L., Mutis A., Quiroz A., Venthur H. (2022). Insights into Chemosensory Proteins from Non-Model Insects: Advances and Perspectives in the Context of Pest Management. Front. Physiol..

[36] Jernigan C.M., Halby R., Gerkin R.C., Sinakevitch I., Locatelli F., Smith B.H. (2020). Experience-dependent tuning of early olfactory processing in the adult honey bee, *Apis mellifera*. J. Exp. Biol..

